# A comprehensive evaluation of life sciences data resources reveals significant accessibility barriers

**DOI:** 10.1038/s41598-025-08731-7

**Published:** 2025-07-02

**Authors:** Sehi L’Yi, Harrison G. Zhang, Andrew P. Mar, Thomas C. Smits, Lawrence Weru, Sofía Rojas, Alexander Lex, Nils Gehlenborg

**Affiliations:** 1 Harvard Medical School, Boston, MA USA; 2 Stanford University, Palo Alto, CA USA; 3 University of California, Berkeley, CA USA; 4 University of Utah, Salt Lake City, UT USA

**Keywords:** Computational biology and bioinformatics, Data publication and archiving, Software, Disability

## Abstract

Individuals with disabilities participate notably less in the scientific workforce. While the reasons for this discrepancy are multifaceted, accessibility of knowledge is likely a factor. In the life sciences, digital resources play an important role in gaining new knowledge and conducting data-driven research. However, there is little data on how accessible essential life sciences resources are for people with disabilities. Our work is the first to comprehensively evaluate the accessibility of life sciences resources. To understand the current state of accessibility of digital data resources in the life sciences, we pose three research questions: (1) What are the most common accessibility issues?; (2) What factors may have contributed to the current state of accessibility?; and (3) What is the potential impact of accessibility issues in real-world use cases? To answer these questions, we collected large-scale accessibility data about two essential resources: data portals (*n* = 3,112) and journal websites (*n* = 5,099). Our analysis shows that many life sciences resources contain severe accessibility issues (74.8% of data portals and 69.1% of journal websites) and are significantly less accessible than US government websites, which we used as a baseline. Focusing on visual impairment, we further conducted a preliminary study to evaluate three data portals in-depth with a blind user, unveiling the practical impact of the identified accessibility issues on common tasks (53.3% success rate), such as data discovery tasks. Based on our results, we find that simply implementing accessibility standards does not guarantee real-world accessibility of life sciences data resources. We believe that our data and analysis results bring insights into how the scientific community can address critical accessibility barriers and increase awareness of accessibility, leading to more inclusive life sciences research and education. Our analysis results are publicly available at http://inscidar.org/.

## Introduction

People with disabilities, such as vision, cognitive, and physical disabilities, encounter barriers in scientific research and education^1^. Researchers and scientific organizations highlighted the importance of addressing this problem and proposed strategies to address the current problems. For example, Swenor and Meeks^1^ highlighted the need for a multifaceted approach, proposing 12 best practices. The Advisory Committee to the Director Working Group on Diversity (ACD WGD) of the National Institutes of Health (NIH) released a report^2^ in 2022, highlighting the importance of data collection related to disabilities to diversify the life sciences workforce. The National Human Genome Research Institute (NHGRI) of NIH announced a long-term action agenda for the next decade^3^ to increase diversity in the genomics workforce, which similarly emphasized the lack of data regarding people with disabilities in the workforce.

There is a discrepancy in the population of individuals with disabilities in the scientific workforce and the US adult population^4^. This indicates potential barriers for individuals with disabilities to join the workforce. There are many factors that are potentially associated with the current barriers, yet digital accessibility is considered one of the important factors^5^. In the life sciences workforce, digital resources, such as data portals and journal websites, play an important role in gaining new knowledge and conducting data-driven research^6^. However, there is little data on how accessible essential life sciences resources are for people with disabilities. For example, 45.2%^7^ of people with visual impairments rely on screen reader assistive technologies, such as NVDA^8^ and JAWS^9^to identify and understand content displayed on the screen. However, multiple studies^10–15^ found that existing digital resources, such as educational websites and PDF files, largely fail to support screen readers, making resources inaccessible to people with visual impairments. There are several studies that evaluated the accessibility of existing resources, such as university and government websites^10–14^ or alternative text (“alt text”) in PDF files^15^. However, such data is largely lacking for the life sciences. To include people with a broad range of disabilities in the life sciences workforce, it is vital to understand and address the digital accessibility issues of existing resources.

Our work is the first of its kind to comprehensively evaluate the accessibility of life sciences resources for people with disabilities, including vision, cognitive, and physical disabilities. The main goal of our study is to understand the current state of accessibility of digital data resources in the life sciences. Specifically, we have the following three research questions. (1) First, what are the most common accessibility issues? Understanding common issues will help us understand how those issues might be addressed. (2) Second, what factors may have contributed to the current state of accessibility? Several studies suggested multiple factors influencing the digital accessibility of websites, such as national regulations^16^. Identifying such potential factors can give guidance on how accessibility can be implemented effectively. (3) Lastly, what is the potential impact of accessibility issues in real-world use cases? Different life sciences websites are designed for different usage scenarios. Therefore, it is vital to understand the actual impact of accessibility issues in the context of individual websites with a user.

We present a large-scale dataset that captures the real-world accessibility of life sciences resources—data portals (*n* = 3,112) and journal websites (*n* = 5,099)—using a computational accessibility testing tool. We conducted a statistical comparison of our data on life sciences resources with US government websites (*n* = 852); websites that need to meet strict legal requirements for accessibility (e.g., Sect. 508 of the Rehabilitation Act)^17^. Focusing on visual impairments, we conducted a preliminary user study, collecting accessibility data of select data portals through manual evaluation with a screen reader user who has no residual vision, unveiling the practical impact of the identified accessibility issues on an individual trying to accomplish common tasks on data portals, such as data discovery tasks.

## Methods

### Computational accessibility evaluation

Following best practices in accessibility research^18^we used both computational and manual approaches to test the accessibility of existing life sciences resources. The computational approach enabled us to collect data about accessibility at scale, which we complemented with a manual evaluation with an actual screen reader user who is totally blind to assess real-world accessibility issues and put our large-scale data into a practical context.

To identify data portals and journal websites for the evaluation, we collected lists of websites from public repositories, i.e., Database Commons^19^ for data portals and Scientific Journal Rankings (SJR)^20^ for journal websites. Of all the data portals (*n* = 6,378) and journal websites (*n* = 27,955), we excluded those that were not considered life sciences resources (e.g., journals in physics) (*n* = 17,774) and that we had issues connecting to (e.g., websites that are no longer available) (*n* = 8,438).

As a result, we selected a total of 3,112 data portals and 5,009 journal websites for our evaluation. In addition to life sciences resources, we collected 852 US government websites as the baseline for our comparative analysis^21^the websites that need to meet strict legal requirements for accessibility (e.g., Sect. 508 of the Rehabilitation Act)^17^. To test the accessibility of the collected websites, we used the Axe accessibility testing tool^22^which showed reliable and comprehensive evaluation results compared to other tools in previous studies^23^. The Axe supports testing various items that are compliant with Sect. 508^24^ and WCAG 2.1^25^ (Web Content Accessibility Guidelines). Using Axe, we collected the accessibility states of web pages, examining whether web pages violate different accessibility issues, such as low contrast ratio, missing alternative text, and empty content. For improved interpretability of the issues defined by Axe^22^ for our study result analysis, we assigned more informative categories to individual issues (Table 1). These categories are related to our first research question, understanding the common issues identified in life sciences resources (e.g., whether issues potentially affect data-driven tasks, such as the lack of descriptions for tables). To assign such categories, all authors iteratively reviewed and discussed individual accessibility issues. This table is defined in a single CSV file, which can be easily updated or replaced by other researchers. Refer to the supplemental data for the full table.

**Table 1:** A list of categories we assigned to individual axe accessibility issues. We reviewed 83 unique accessibility issues observed in our study with axe^22^ and created the categories introduced here for our analysis.

Category	Label Description	Values
Overall Impact	The overall impact of a given issue in the context of life sciences data resources. This label summarizes the four categories below (Criticality, WCAG Level, Difficulty to Fix, and Data Related). For example, if an issue is data-related, critical, difficult to fix, and related to WCAG Level A, the issue is considered to severely impact user tasks.	Severe Moderate Minor
Criticality	This category describes whether the issue can entirely block users from performing their tasks.	Critical Less Critical
WCAG Level	This category represents the minimum WCAG^25^ level of conformance that is related to a given issue. An issue with Level A indicates that it is the most essential issue for the success criteria. An issue with Level AA in our study represents that it is an important issue for enhancing accessibility, but it is not part of Level A. Therefore, issues with Level A are the highest priority.	A AA
Difficulty to Fix in Post-Deployment	This captures how difficult it is to fix a given issue after the deployment (“post-deployment”), such as addressing issues on already deployed data portals using browser extensions.	Difficult Moderate Easy
Data Related	This label describes whether the issue is related to perception or interaction with data (e.g., a table in the wrong structure).	Yes No
Missing-label Related	This category captures whether the issue is related to missing labels (e.g., missing alternative text of images).	Yes No

In order to compare the Axe results of individual websites, we calculated the failure rate (FR) per website, which is one of the most widely used metrics, taking into account the size and complexity of websites^26^.

1 $$\:{I}_{p}=\frac{{B}_{p}}{{P}_{p}}$$.

The failure rate of a web page $\:{I}_{p}$ is defined as the ratio between the actual points of accessibility violations $\:{B}_{p}$ and the potential points of violations $\:{P}_{p}$. A completely accessible website thus has a failure rate of 0 (i.e., none of the elements of the page have any violations), whereas a completely inaccessible website has a failure rate of 1 (i.e., all of the elements on the page have all potential violations).

We merged the accessibility evaluation results of individual resources with their contextual information provided from the original repositories (i.e., Database Commons and SJR). This includes geospatial dimensions (e.g., country and city), temporal dimensions (e.g., year founded), and impact scores (e.g., citation counts) of data portals and journals.

We used Python (3.10.13) with Altair^27^ (5.1.2) in Jupyter Notebooks^28^ to collect, manipulate, and visualize data. The accessibility reports are collected using the 4.9.1 version of Axe-core.

### Statistical analysis

We performed statistical analyses on the accessibility states to find significant differences across subgroups: resource types (i.e., data portals, journal websites, and US government websites), countries, continents, and hosting institutions. We performed Dersimonian and Laird’s random-effects meta-analyses^29^ to summarize the failure rates of the websites within subgroups and used a mixed-effects model to account for potential heterogeneity and differences in underlying distributions of failure rates between different websites. The failure rate for a given journal website, government website, or data portal was counted as a single statistic during meta-analysis, and standard errors were estimated using the total number of checks performed for a given page. We assessed significant differences between subgroups of websites by comparing 95% confidence intervals estimated from the meta-analysis.

### Manual accessibility evaluation

To see the potential impact of the identified accessibility issues on actual users, we additionally conducted a preliminary manual evaluation using three data portals—cBioPortal^30^HuBMAP Data Portal^31^and ENCODE Data Portal^32^—that are widely used. A co-author of the paper who is blind (hereafter, “a screen reader user”) tested data portals using the JAWS screen reader assistive technology^9^. The first author led the user studies. The manual evaluation was conducted remotely via Zoom. The overall procedure of the manual evaluation was designed based on Pernice and Nielsen^33^. We conducted three study sessions on different days, evaluating a single data portal in each session. The session lasted between 1.5 and 2.5 h.

In each session, the screen reader user was asked to perform ten tasks and given 5 min to complete each task. We designed ten user tasks for each data portal. The tasks are adopted from the Use Case Library of Common Fund Data Ecosystem (CFDE)^34^which provides high-level objectives and tasks that need to be considered when designing life sciences data repositories. We initially selected 10 high-level tasks from this library that are commonly supported in the three selected data repositories. We then made the tasks explicit to reflect the context of each data repository. Example tasks include finding specific datasets by applying filters (e.g., “Find kidney datasets for donors over the age of 65.”) and identifying linked publications (e.g., “Find a list of publications that used data in this data portal. How many peer-reviewed papers are there?”). Refer to the Supplemental Note to find all tasks for the three data portals. After either finishing each task or exceeding the 5-minute time limit, the user was asked to answer three questions on a 7-point Likert scale: (1) How confident are you that you performed the given task accurately?; (2) How satisfying was it to use the website to perform the task?; and (3) How frustrating was it to use the website for a given task? The user was asked to elaborate on why they came up with specific scores. After repeating this process for all ten tasks, we finished the session with an interview to collect their overall impression of the accessibility of the given data portal. Through this manual testing, we collected the time taken and the success of each task, as well as scores for three subjective questions.

The protocol of the manual evaluation was reviewed by the Institutional Review Board of Harvard University (IRB24-1220), and it was determined that this study meets the criteria for exemption under federal regulations. All experimental protocols were approved by the Harvard IRB. All methods were carried out in accordance with relevant guidelines and regulations. Informed consent was obtained from the participant.

## Results

### Most common accessibility issues

**Fig. 1:**
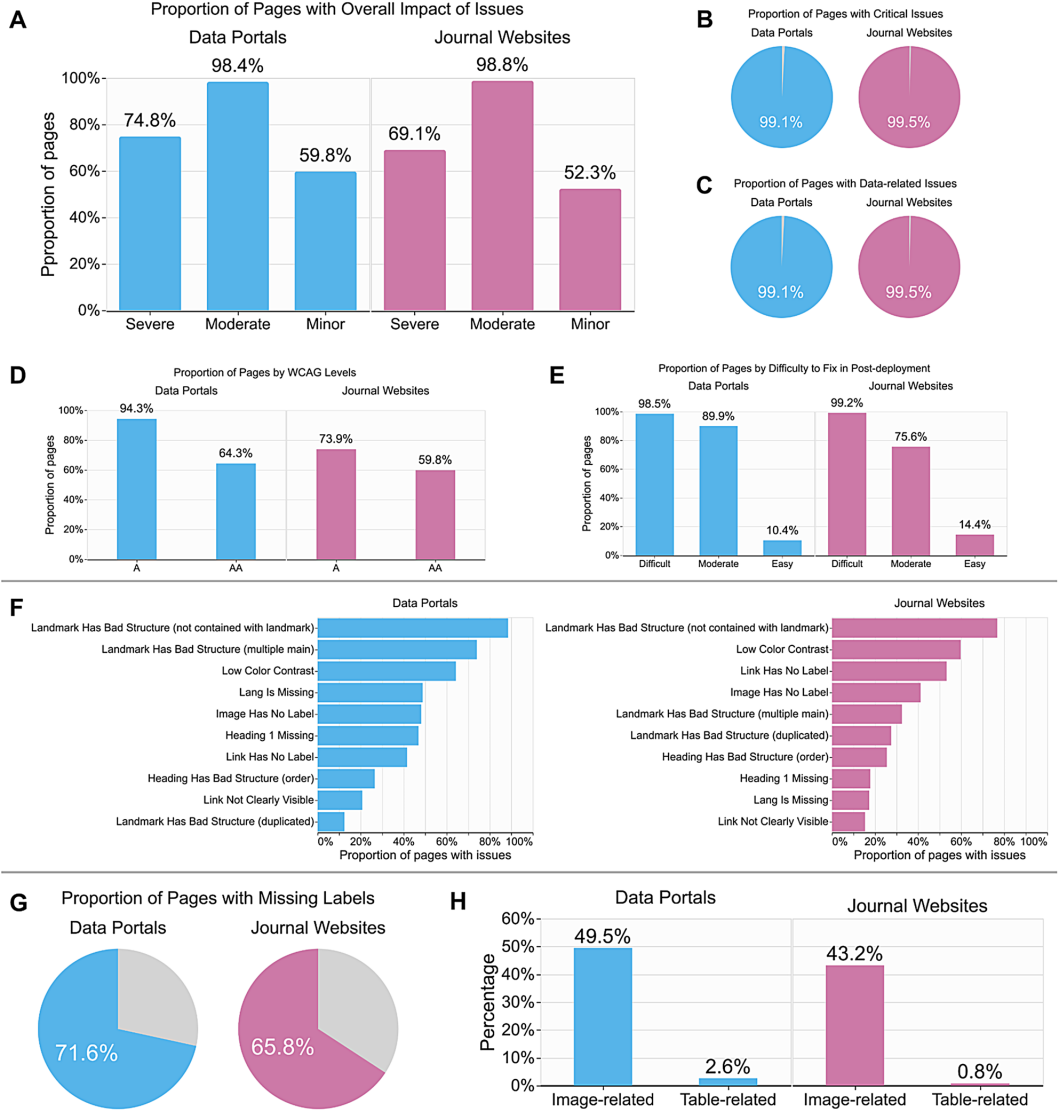
Accessibility issues found on life sciences websites: Proportion of pages with issues in terms of their (**A**) overall impact, (**B**) criticality, i.e., whether the issue is likely to block users performing tasks entirely, (**C**) whether they are related to the perception of and interaction with data (e.g., issues in tables), (**D**) WCAG levels^25^and (**E**) difficulty to fix in post-deployment (e.g., using browser extensions to work around accessibility issues in already deployed websites); (**F**) Top 10 most frequently observed accessibility issues. The proportion of pages with (**G**) missing labels and (**H**) image-related and table-related issues.

**Fig. 2:**
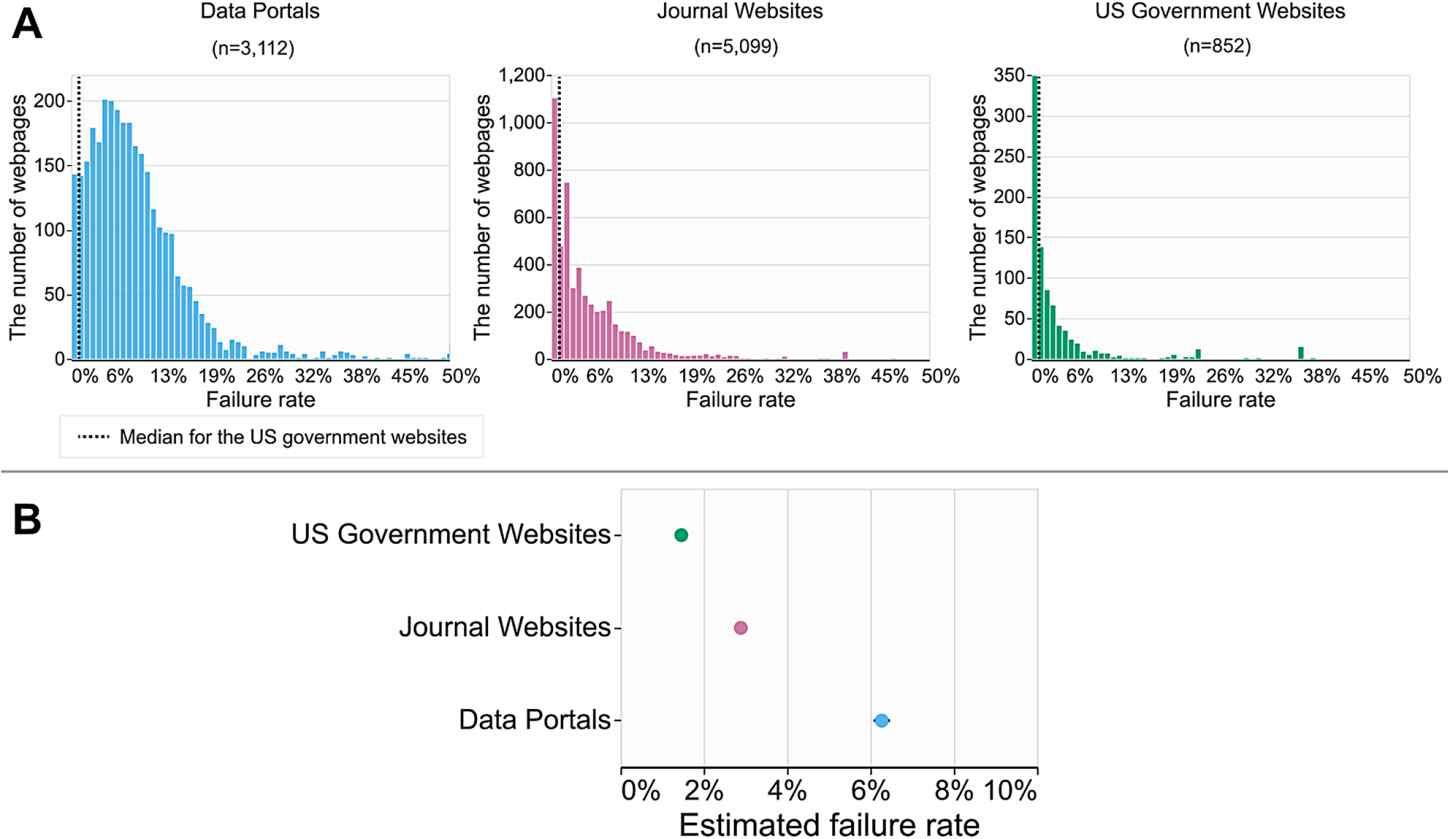
The failure rates of data portals, journal websites, and US government websites. (**A**) The distribution of failure rates of individual web pages is shown in histograms. The y-axis represents the number of web pages with the corresponding failure rates (x-axis). The x-axis is truncated to 50% failure rates to make the distributions readable in the charts. There were 46 web pages with failure rates higher than 50% (32 data portals, 9 journals, and 5 US government websites). The median failure rate of the US government websites (1.1%) is drawn as dotted vertical lines for a standard reference. (**B**) The *estimated* failure rates of three groups of websites based on statistical analysis (refer to the Methods section) are shown in dot plots. The error bars represent the 95% confidence intervals. The failure rate is a metric that measures the accessibility issues of a website while considering its size. For example, a failure rate of 50% indicates that of all possible accessibility issues, a site actually fails on 50% of the issues (refer to the Methods section).

**Fig. 3:**
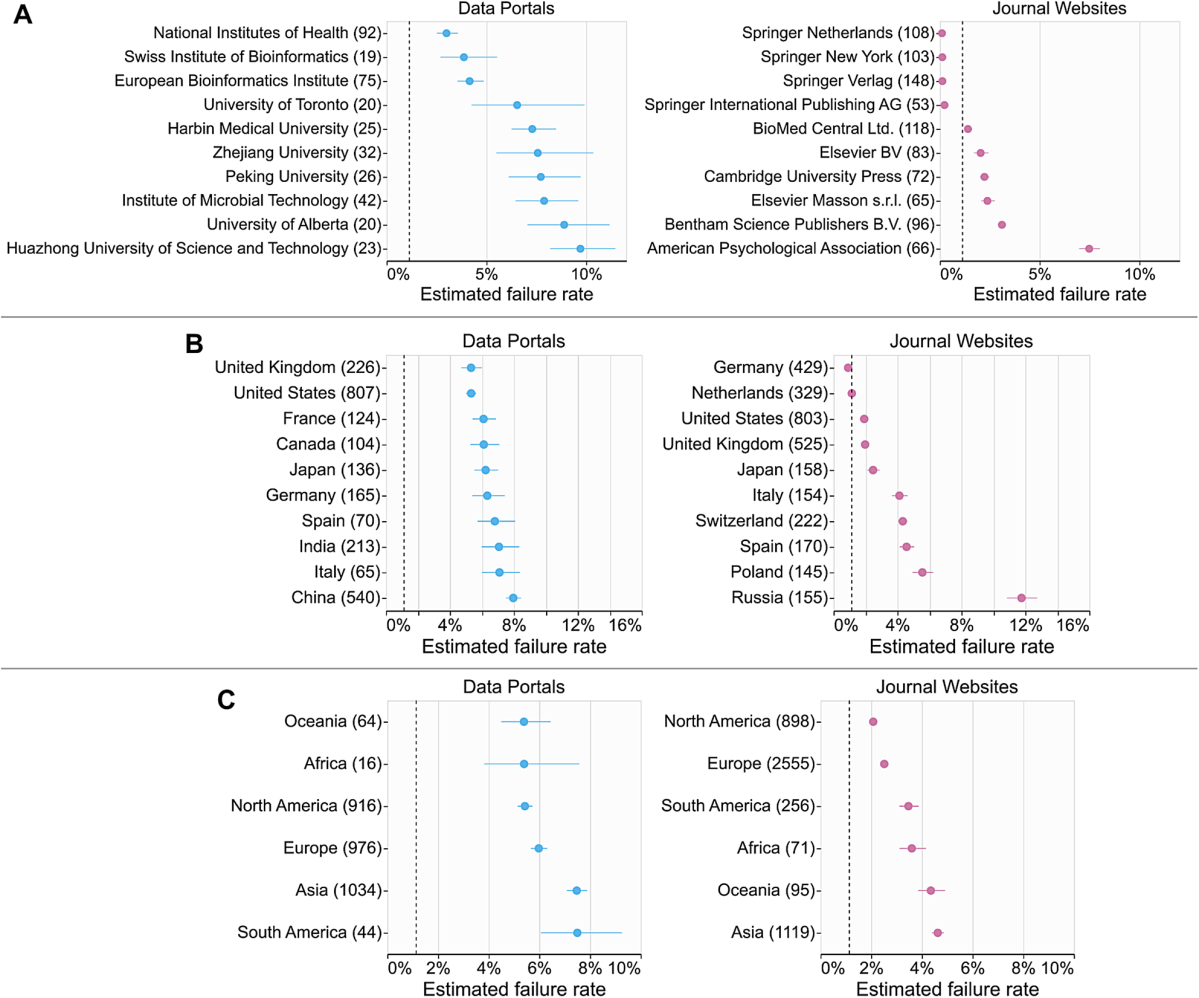
The estimated failure rates of data portals and journal websites grouped by their (**A**) hosting institutions and publishers, respectively, (**B**) countries, and (**C**) continents. The error bars represent the 95% confidence intervals. The numeric values inside brackets in the y-axis represent the sample size n of the corresponding groups. The US government websites’ median failure rate (1.1%) is drawn as vertical dashed lines for reference. Only the top 10 hosting institutions (out of 141 for data portals and 121 journals) and the top 10 countries (out of 35 for data portals and 66 for journals) with the largest sample sizes (n) are shown.

We analyzed accessibility issues after categorizing them based on several criteria (refer to the Methods section for details). Overall, more than half of the life sciences websites—74.8% data portals and 69.1% journal websites—contain “severe” accessibility issues (Fig. 1A). The severe accessibility issues are identified in our analysis (refer to Methods) by testing several criteria, such as their criticality in performing user tasks and difficulty to fix (Table 1). Most importantly, almost all websites contain issues that (1) can critically block users from performing data-related tasks (Fig. 1B and C), (2) are related to minimum WCAG requirements (Fig. 1D), and (3) cannot be easily fixed after the deployment of the websites (Fig. 1E) (e.g., using a browser extension to fix issues on existing websites). A typical example is the absence of required labels (Fig. 1G), such as missing alternative text (or “alt text”) for images and links, which is one of the most frequently identified accessibility issues on data portals and journal websites (Fig. 1F). Other common types of issues are ill-structured web pages (e.g., broken heading structure that makes it difficult to navigate a web page with a keyboard and a screen reader) and low color contrast (e.g., links that are hard to distinguish from regular text for low-vision users).

Since life sciences data and information are frequently represented in images and tables (e.g., dataset tables in cBioPortal^30^), we explored how many issues relate to images and tables on web pages (Fig. 1H). Our data shows that nearly half of the data portals (49.5%) and one-third of the journal websites (43.2%) had image-related accessibility issues. With regards to tables, 2.6% of data portals and 0.8% of journal websites had accessibility issues, a much lower rate than images. Common table-related issues are missing labels for table headers (e.g., column names), which make it difficult for users with visual impairments to navigate and perceive a large data table efficiently. Since navigating data tables is considered one of the most important tasks for data portal end users^34^even a few accessibility issues on tables can prevent people with visual impairments from accessing essential information.

### Current state of life sciences website accessibility

Overall, our results show that life sciences websites largely fail to meet accessibility standards. The distribution of the failure rates of individual web pages (Fig. 2) shows that data portals are skewed the most toward the higher failure rates compared to the other two resources. Our statistical analysis results reveal that the failure rates of life sciences websites are significantly higher than those of US government websites. Government websites showed the lowest estimated failure rates (1.5%), followed by journal websites (2.9%). Data portals showed the highest estimated failure rates (6.3%), more than twice the rate of journal websites and four times the rate of government websites. Notably, almost all data portals (93.6%) showed higher failure rates than the US government websites’ median failure rates (dotted lines in Fig. 1). These include widely used data portals, including cBioPortal^30^ (19.3%), KEGG^35^ (3.7%), and ENCODE^32^ (2%).

In summary, life sciences websites significantly fail to meet accessibility standards, which leads to critical barriers for people with disabilities in accessing and using these essential resources for life sciences education and research.

### Factors influencing accessibility issues

#### Hosting institutions

We further analyzed the collected data at the aggregated level using several key categories. We first see whether hosting institutions of life sciences websites have affected accessibility (Fig. 3A). Our data shows that some institutions’s pages vary a lot with respect to failure rates, while pages hosted by others have more consistent failure rates (error bars in Fig. 3A). Interestingly, data portals hosted by universities generally showed higher failure rates with higher variability (as seen with wider confidence intervals in Fig. 3A), while data portals hosted by (inter)national institutions (i.e., the three rows on the top), such as European Bioinformatics Institute (EMBL-EBI) and National Institutes of Health (NIH), showed significantly lower and less variable failure rates.

#### Countries and continents

Despite the notable differences between life sciences data portals and journal websites in terms of their contents and use cases, failure rates by geographic region were overall consistent between the two groups (Fig. 3B–C). In both groups, websites from China and India showed significantly higher failure rates than the ones from United States and the United Kingdom (Fig. 3B). Similarly, at the continent level, websites from Asia showed significantly higher failure rates compared to the ones from Europe, Oceania, and North America (Fig. 3C). This trend aligns with a recent large-scale accessibility study in general websites^36^where websites in English showed better accessibility compared to websites in other languages, such as Mandarin. Notably, none of the countries and continents showed better accessibility results than the US government websites (dotted lines in Fig. 3B–C), except for journal websites by Germany-based publishers (e.g., *Biological Chemistry*, ISSN: 1437–4315, by Walter de Gruyter). National disability policies are considered to play an essential role in digital accessibility, and our data shows a consistent pattern. In a cross-country accessibility study^16^several Asian countries, including China, are considered to have “weak” disability policies. In contrast, European countries, including Germany and the United Kingdom, are considered to have “strong” policies. In 2002, Germany enacted the ordinance on barrier-free information technology (BITV), which extends the Web Content Accessibility Guidelines (WCAG). This mandates that all public websites in Germany meet this accessibility standard. In line with this, journal websites from Germany showed significantly lower failure rates than other countries.

### Real-world impact of accessibility issues

**Fig. 4:**
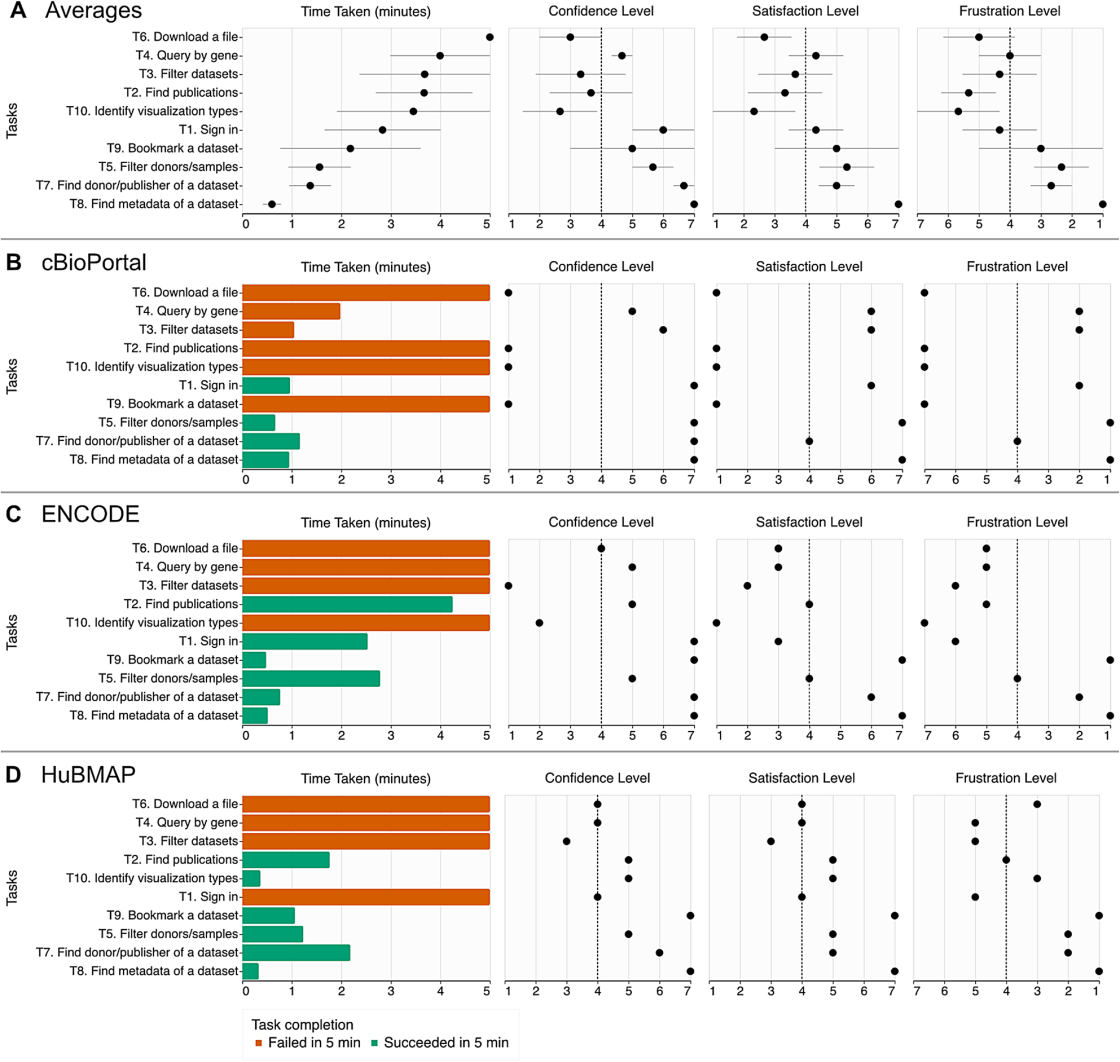
Results of manual evaluation of three data portals^30–32^ with a blind screen reader user. The user performed ten typical tasks on each data portal and gave subjective scores about their confidence, satisfaction, and frustration levels for each task. (**A**) The three results are aggregated with mean completion time and subjective responses, where error bars represent standard deviation. (**B**–**D**) Individual results of three data portals are shown: (**B**) cBioPortal^30^, (**C**) ENCODE Data Portal^32^, and (**D**) HuBMAP Data Portal^31^. The HuBMAP Data Portal is developed and maintained by the research group led by the senior author of this paper. Note that the purpose of the manual evaluation is not to compare data portals but instead to understand how accessibility issues identified by computational approaches influence the actual use cases.

Our preliminary user evaluation of selected important data portals with a blind user complements the computational evaluation results, showing the potential influence of the accessibility issues we identified in real-world use cases. The study participant performed a total of 30 tasks in three data portals^30–32^which had failure rates of 19.3% (cBioPortal), 2% (ENCODE), and 8.8% (HuBMAP) in our computational evaluation. Refer to the Methods section for the manual evaluation protocol. The participant successfully performed only about half of the tasks (16 out of 30, 53.3%), spending 2.8 min out of a maximum of 5 min per task on average (Fig. 4). The participant, on average, felt that they were somewhat confident (4.8 out of 7), somewhat satisfied (4.3), and somewhat not frustrated (3.76) with the data portals for each task, where their subjective responses vary across tasks and data portals (Fig. 4A—B). Overall, the participant was able to find metadata of a given dataset or study in all data portals (T5, T7, T8). However, the participant consistently failed to find and download specific datasets (T3, T4, T6).

Through this study, we found evidence of how different types of accessibility issues prevent users from performing typical data portal tasks. For example, the most common and critical accessibility issues hindering the completion of the tasks were related to buttons, links, and images without labels (e.g., alt-texts). Developers usually have to provide such labels when implementing data portals. Sometimes, data portals use icons for buttons without alt text, making it extremely difficult for the user to identify buttons for downloading files (T6) or bookmarking datasets (T9). Sometimes, users can still understand the function of the mislabeled components through trial and error. However, the complexity of pages in data portals made it very difficult for the participant to guess the functionalities of buttons with missing labels.

We also observed several barriers that the participant encountered that the computational evaluation could not identify. For example, the participant had to apply several filters (e.g., assay types or donor ages) using checkboxes, search fields, and even sometimes sliders to search for specific datasets (T3, T4). The participant was able to use these components correctly for some data portals. However, the user found it difficult to grasp all the filters applied in the data portals. This often made the participant apply the wrong filters, leading to inaccurate search results. Another example is that the participant consistently found it confusing to use the auto-complete features of keyword search since the user did not perceive suggested keywords and did not understand that the participant had to select one of the suggested keywords to proceed with the search. These examples show how specific tasks in data portals could be challenging to perform even when accessibility standards are met.

## Discussion

### Comparison to prior accessibility evaluations

There have been many studies that evaluated the digital accessibility of public websites. Predominant types of websites evaluated include universities, government institutions, and library websites. For example, Van Loon and McCann evaluated 25 STEM databases using the WAVE browser extension^37^. Liu et al. evaluated the 100 library websites of US universities using both WAVE and AChecker^38^. Overcoming the limitation of using automated tools only, Southwell and Slater evaluated 68 library websites of US colleges and universities, both using WAVE and through manual testing with individuals without disabilities^39^. Although many accessibility user studies were conducted with able-bodied participants, conclusions and insights from such studies can be inaccurate and misleading^40^. To better understand practical barriers from real users, Mulliken^41^ conducted user interviews with 18 blind library website users and highlighted the significantly longer time required for blind users compared with sighted users. This result is consistent with our work, as our manual evaluation showed that a study participant was not able to complete simple tasks within five minutes (e.g., signing in to a website or clicking on a download button). Boellstorff^42^ conducted more than two years of fieldwork to describe how digital media can be disabling through its design.

These studies focused on only a small number of websites, limiting comprehensive insights into existing websites. More recent studies conducted a large-scale evaluation by testing more than 1 K websites. Burgstahler et al.^43^ analyzed country-wise accessibility states of 8,557 higher education websites and 6,872 national government websites using an automated tool. The results showed the potential impact of national laws and policies on digital accessibility. Kimmons^44^ evaluated 3,141 college and university websites in the US in 2017. Another study^45^ analyzed an even larger scale analysis, assessing the accessibility of 2.7 million Italian public administration websites. A more recent study^46^ analyzed nearly three million web pages using an automated tool and found only a very small number of web pages (0.5%) had no errors. While these studies provide a comprehensive overview of the accessibility states in the wild, their insights cannot be generalized to life sciences data resources. Moreover, such large-scale studies commonly lack the insights from actual screen reader users. Due to the limitation of data collected only through automated tools, it is recommended to conduct both the automated and manual evaluation approaches^18^.

There are several studies that evaluated public websites using both approaches. For example, Yoon et al.^47^ evaluated the accessibility of six public library websites based on both a usability study with six individuals with visual impairments and using the AChecker automated tool. The authors found that the automated testing does not necessarily find practical errors that screen reader users encounter, confirming prior findings by Rømen and Svanæs^48^. Our study also confirms this, where a data repository with a low failure rate still leads to a low task completion rate with an actual screen reader user.

To the best of our knowledge, there are no prior studies that evaluated the digital accessibility of life sciences data resources designed for researchers. Most work in life sciences has focused on digital health accessibility^49^ or education resource accessibility^5^.

### Benefits of centralized development for accessibility

In our evaluation results, the centralized development and maintenance of data resources at national or international institutions may have resulted in more consistent and better accessibility of their life sciences websites^50^. For instance, EMBL-EBI has implemented and maintains a website construction framework—Visual Framework^51^—for their life sciences websites. This framework incorporates accessibility guidelines and best practices as built-in features, which have been iteratively improved over time and are consistently used across websites that the institution maintains. Since learning and implementing accessibility standards is considered notably challenging and time-consuming for developers, using a shareable framework with accessibility standards built in seems to be largely beneficial in supporting better and consistent accessibility. A similar example to improve accessibility of research papers would be having clear accessibility guidelines in journals, as a previous study found that none of the 289 journals the authors reviewed had alt text guidelines for Figures^52^.

### Implications for real-world accessibility

As our manual evaluation showed, our computational accessibility results underestimate the real-world accessibility issues for actual users. This means that even though our computational results showed a low failure rate (e.g., 1%) for certain life sciences (data) resources, it does not mean that they are overall (e.g., 99%) accessible to users with disabilities. The failure rate has previously been noted to underestimate the inaccessibility of websites in a metric validity study, where inaccessible websites had very low failure rates ranging from 0.0704 to 0.271^26^. As such, we should reconsider the cut-off value for which we deem a website inaccessible.

The accessibility metrics, like failure rates, do not take into account the criticality of the component related to the task. There is no metric that can be evaluated automatically, as the task and, therefore, critical components vary. For example, failures to implement accessibility standards on a few critical components of a web page (e.g., a figure containing important life sciences concepts or a data table containing data essential for data-driven research) can still make the web page entirely useless for users with a disability. In our manual evaluation, many tasks that our participant could not complete successfully, such as downloading files and bookmarking datasets, were caused by a single or a handful of inaccessible components on the website. Hence, automatic approaches can only show an incomplete picture, and conducting user studies with people with disabilities is required to better assess real-world accessibility.

### Going beyond existing accessibility standards

Our manual evaluation showed that merely implementing accessibility standards does not guarantee that users can successfully perform typical tasks in data portals. For example, even though two of the data portals in the manual evaluation implemented accessibility standards for interactive components well (e.g., providing readable labels to buttons, checkboxes, and sliders), the participant found it difficult to interact with large datasets (e.g., applying proper filters to select datasets of interest). This is consistent with findings from previous accessibility evaluations that compared manual and automatic evaluation results^47,48^. Therefore, we think that making life sciences data accessible to people with disabilities requires more than simply following general accessibility standards; it requires more studies and specialized solutions in the life sciences field. For example, given that each data portal can have different use cases in mind, it is vital to include people with disabilities in the design process (i.e., co-design with people with disability) to accurately support accessibility in different contexts.

### Limitations

A limitation of our work is that we evaluated only the landing pages of life sciences resources in our computational accessibility testing. In future work, we will focus on reliably identifying common types of subpages (search page, browse page, data page, article page, etc.) of resources at scale. However, given the vital role of landing pages (i.e., people need to first access the landing pages to reach other subpages), we believe that our analysis of home pages provides essential insights into the extent to which current life sciences resources are accessible. Moreover, we mitigate the impact of this limitation on our overall recommendations by conducting manual evaluations with a screen reader user by testing multiple meaningful pages per website (e.g., data page, detail page, and documentation).

### Future work

We envision several future directions that extend our research questions: deepening our understanding of (1) common accessibility issues for visualizations, (2) barriers of developers of life sciences data resources for learning and implementing accessibility standards, and (3) the comprehensive real-world impact of accessibility issues with a larger number of users.

Given the importance of visualization in data repositories, we tried to see in the manual evaluation whether a screen reader user can identify the essential information of visualizations embedded in data portals (i.e., visualization types). However, many different accessibility aspects of visualization need to be tested to ensure its usefulness to actual users. To better understand the accessibility of data visualization in life sciences, we will include additional scientific tools for using, exploring, and analyzing data, such as genome browsers. Also, to test the accessibility more comprehensively, we will adopt accessibility standards developed specifically for data visualizations, such as Chartability^53^.

The role of developers is crucial for making websites accessible. However, we lack our understanding of the current barriers of developers of life sciences data websites for learning and implementing accessibility standards. We will conduct user interviews with developers for data repositories and visualization tools in life sciences to unveil barriers and identify potential ways to address them.

Lastly, to have a more comprehensive real-world accessibility of life sciences websites, we will conduct a series of user studies with not only individuals with visual impairments but also people with other disabilities, such as motor impairments that impact the use of input devices (e.g., keyboard-only interactions). This will enable our community to understand common and unique accessibility problems that individuals with different disabilities encounter when interacting with life sciences data websites.

## Electronic supplementary material

Below is the link to the electronic supplementary material.

Supplementary Material 1

## Data Availability

All data used in our study are available through Figshare (DOI: 10.6084/m9.figshare.26801032.v1). All source code to collect, analyze, and visualize data is publicly available on GitHub at https://github.com/inscidar/analysis-notebooks.
