# Supplementary material for: A comprehensive evaluation of life sciences data resources reveals significant accessibility barriers
Source: Sci Rep. 2025 Jul 2;15:23676. doi: 10.1038/s41598-025-08731-7 (PMC12222754; doi:10.1038/s41598-025-08731-7)
Supplement: Supplementary file 1 — Supplementary Material 1 [file 41598_2025_8731_MOESM1_ESM.pdf]

A comprehensive evaluation of life sciences data resources reveals significant accessibility barriers

Sehi L’Yi[1], Harrison G. Zhang[2], Andrew P. Mar[1,3], Thomas C. Smits[1], Lawrence Weru[1], Sofía Rojas[1], Alexander Lex[4], Nils Gehlenborg[1]

[1]Harvard Medical School, MA, USA

[2]Stanford University, CA, USA

[3]University of California, Berkeley, CA, USA

[4]University of Utah, UT, USA

Supplemental Note

Tasks for Manual User Evaluation

Portal	ID	Task
HuBMAP	1	Sign in to the HuBMAP data portal
HuBMAP	2	Find a list of publications that used HuBMAP data. How many peer-reviewed papers are there?
HuBMAP	3	Find kidney datasets for donors over the age of 65
HuBMAP	4	Query data for a gene named CTPS1. Use parameters of Gene Expression (RNA), Minimum Expression Level of 10, and Minimum Cell Percentage of 2. What is the HuBMAP ID of the first data appearing in the result?
HuBMAP	5	Find male donors that are between 35 and 65 years old with a BMI (Body Mass Index) above 18
HuBMAP	6	For a given dataset, download a file for a dataset
HuBMAP	7	For a given dataset, go to a donor page to find donor metadata
HuBMAP	8	For a given dataset, find contributors to the dataset
HuBMAP	9	For a given dataset, save or bookmark the data on the page and see it in the account.
HuBMAP	10	Find a visualization on a dataset page. Identify what kinds of visualization there are.
ENCODE	1	Sign in to the data portal
ENCODE	2	Find a list of publications of the ENCODE project. Can you find the title of the first publication?
ENCODE	3	Find kidney datasets for donors who are male
ENCODE	4	Search by region (gene name: CTCF) for mouse genomes (Mus Musculus).
ENCODE	5	Find a page for single-cell experiments and find kidney datasets. How many datasets are there?
ENCODE	6	For a given dataset, download a file with a file extension of BEDPE.
ENCODE	7	For a given dataset, go to an award page to find award metadata. Can you identify who is the primary investigator?
ENCODE	8	For a given dataset, find the Biosample Type of the dataset
ENCODE	9	For a given dataset, add the dataset to your cart
ENCODE	10	Find a visualization on a summary page. Identify what kinds of visualization there are.
cBioPortal	1	Sign in to the data portal
cBioPortal	2	Find a list of publications of the cBioPortal project. Can you find the title of the first publication?

cBioPortal	3	Filter datasets (here, studies) that are related to Kidney.
cBioPortal	4	Can you search datasets by gene (gene name: CTCF)?
cBioPortal	5	Can you filter datasets by data type? Select datasets with "Mutations" data type.
cBioPortal	6	For a given study, download a file.
cBioPortal	7	For a given dataset, find the gender of the donor. You may go to another page for this task.
cBioPortal	8	For a given dataset, find the assay type.
cBioPortal	9	For a given dataset, add the dataset to your bookmark.
cBioPortal	10	Find visualizations on the Summary page. Identify what kinds of visualization there are.
